# Negative Survival Impact of High Radiation Doses to Neural Stem Cells Niches in an IDH-Wild-Type Glioblastoma Population

**DOI:** 10.3389/fonc.2018.00426

**Published:** 2018-10-04

**Authors:** Xavier Muracciole, Wassim El-amine, Emmeline Tabouret, Mohamed Boucekine, Anne Barlier, Gregorio Petrirena, Tovo Harivony, Laetitia Solignac, Olivier L. Chinot, Nicolas Macagno, Dominique Figarella-Branger, Laetitia Padovani

**Affiliations:** ^1^Radiotherapy Department, Assistance Publique des Hôpitaux de Marseille, Marseille, France; ^2^Neuro-Oncology Department, Assistance Publique des Hôpitaux de Marseille, Marseille, France; ^3^Angiogenesis and Micro Environnment UMR 911 CRO2, Aix-Marseille University, Marseille, France; ^4^Unity of Research EA3279, Aix-Marseille Université, Marseille, France; ^5^Molecular Biology and Oncogenetics Department, Assistance Publique des Hôpitaux de Marseille, Marseille, France; ^6^Neuropathology Department, Assistance Publique des Hôpitaux de Marseille, Marseille, France; ^7^CRCM INSERM UMR1068, CNRS UMR7258 AMU UM105, Genome Instability and Carcinogenesis, Institut Paoli-Calmettes, Marseille, France

**Keywords:** glioblastoma, MGMT, stem neural niches, radiotherapy, dose, survival impact, neural niches

## Abstract

**Aims:** Assess the impact of radiation doses to neural stem cell (NSC) niches in patients with IDH-wild-type glioblastoma.

**Materials and Methods:** Fifty patients were included in the study. NSC niches [SubVentricular Zone (SVZ) and Sub Granular Zone (SGZ)] were contoured by fusing CT scans and pre-therapy MRI, Tumor location defined ipsilateral and contralateral SVZ and SGZ. Prognostic significance of clinical, biological and dosimetric parameters were examined. We generated a Recursive Partitioning Analysis (RPA) model with independent prognostic classes.

**Results:** Median follow-up: 23.8 months. Event free and overall survival (OS): 10 and 19.1 months. Incomplete surgery, PTV (planning target volume), ipsilateral SVZ or NSC niche mean dose > 57.4 Gy, contralateral NSC niche mean dose > 35 Gy and bilateral NSC niche mean dose > 44 Gy were significantly correlated with reduced OS. Only EGFR amplification was an independent prognostic factor (*p* = 0.019) for OS. RPA generated independent risk groups: 1 (low risk): [ipsilateral NSC mean dose (INMD) < 58.01 Gy and methylated MGMT promoter], 2: (INMD < 58.01 Gy and unmethylated MGMT promoter and contralateral SVZ mean dose < 18.6 Gy; *p* = 0.43), 3: (INMD < 58.01 Gy and unmethylated MGMT promoter and contralateral SVZ mean dose > 18.6 Gy; *p* = 0.002) and 4: (very high risk) (INMD > 58.01 Gy; *p* < 0.001).

**Conclusion:** High radiation doses to ipsilateral NSC and contralateral SVZ could have a negative impact on overall survival in IDH-wild-type glioblastoma population.

## Introduction

Glioblastomas are tumors which currently have a terrible prognosi Isocitrate Dehydrogenase (IDH)-wild-type glioblastoma (90%) is most often a primary or *de novo* tumor on the contrary to IDH-mutant glioblastoma (10%) which mostly corresponds to so called “secondary glioblastoma,” resulting from the progression of a low-grade glioma (according to the new 2016 World Health Organization (WHO) classification)(1). The Stupp protocol, which consists of radiotherapy and concomitant chemotherapy with temozolomide has nevertheless succeeded in increasing the 5-year survival rate from 1.9 to 10.4% (2). Prognostic factors of patients with glioblastoma are clinical [age at diagnosis, general state of health and the Karnofsky Performance Status (KPS)], histomolecular [IDH mutation, O^6^-methylguanine-DNA-methyltransferase (MGMT) promoter methylation status] and therapeutical [extent of surgical resection] (3).

In 1998, Eriksson et al. reported the existence of neurogenesis from normal neural stem cells (NSC) in the adult human brain with a capacity to migrate, and to participate actively in brain tissue repair (4). These can be found in two main zones or “neural niches” in the brain: the Sub Ventricular Zone (SVZ) located between the lateral ventricles and the striatal parenchyma, and the Sub Granular Zone (SGZ) confined to the hippocampal dentate gyrus. Part of these cells, initially found in the neural niches, could then migrate toward more peripheral zones of the brain parenchyma leading to more mature cells and the formation of the tumor mass. Cancer Stem Cells (CSC) deriving from these NSC could lead to the formation of glioblastoma. Neural niches therefore harbor both NSC and CSC which directly participate in tumorogenesis (5). The radioresistance of CSC has been clearly described, (6, 7) especially when they were in neural niches (8, 9). This radioresistance is reported as one of the reasons of dramatic prognosis for glioblastoma. However, different retrospective studies regarding this hypothesis have led to contradictory results on the prognostic impact of the total radiation dose to these neural niches (10–12).

Here, we performed a retrospective analysis of patients irradiated for an IDH-wild-type glioblastoma to investigate the impact of SGZ and SVZ radiation on progression-free survival (PFS) and overall survival (OS) in this population of patients. We employ RPA to divide IDH-wild type glioblastoma patients into prognostic groups using clinical molecular and dosimetric data.

## Patients and methods

### Patients

We carried out a single-center retrospective study including all of the patients newly diagnosed with IDH-wild-type glioblastoma and treated with radiotherapy and concomitant chemotherapy according to the Stupp protocol in the University Hospital La Timone, Marseille, France between January 2008 and January 2013. All patients signed a consent form before inclusion in the study and are included in the SIRIC (Site de Rechcerche Integrée en Cancérologie) cohort.

For all patients, the anatomopathology diagnosis was reviewed and immunohistochemistry using anti-IDH1R132H was performed. If patients were less than 55 years old, we tested for IDH gene mutations and all of the patients were negative.

Inclusion criteria for the study were:

- age > 18 years old
- proved histological diagnosis of IDH-wild-type glioblastoma
- pre-therapy magnetic resonance (MR) examination available

Patients were excluded for the following reasons: different radiotherapy protocols (hypofractionated), radiotherapy without chemotherapy prior to the Stupp protocol and patients without MR scans.

### Delineation and treatment planning

Dosimetric data for all of the patients were collected from the computer base archives. Target volumes were defined according to European Association of Neuro-Oncology (EANO) guidelines (GTV, gross target volume; CTV, clinical target volume; PTV, planning target volume). All of the patients received a total radiation dose of 60 Gy in 30 fractions of 2 Gy on PTV. Treatment plans were drawn up using the Pinnacle planning system.

**Figure 1:**
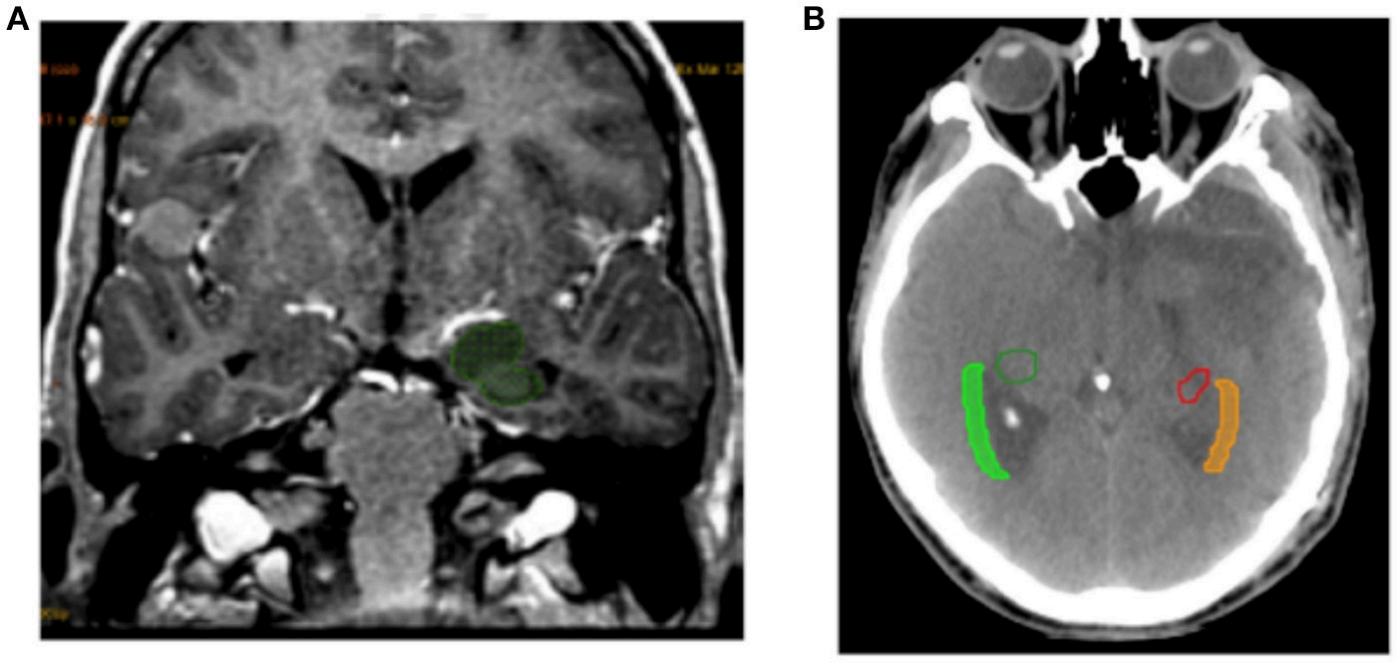
Example of delineation. **(A)** Example of hippocampus delineation included SGZ. **(B)** Example of SVZ delineation from the lateral wall of the lateral ventricles with a margin of 5 mm.

The SVZ was defined on the CT scan as the lateral wall of the lateral ventricles with a margin of 5 mm. The SGZ was located in the hippocampus by fusing the images from the CT scan and the pre-therapy MR scan (Figure 1). Delineation of the hippocampus was based on Radiation Therapy Oncology Group (RTOG) guidelines (13).

Tumor location defined the laterality of the studied volumes (SVZ and SGZ). When the tumor crossed the median line its geometrical center was used to define laterality. Thus the SVZ and SGZ were separated into ipsilateral or contralateral volumes.

Furthermore, initial tumor location was divided into 4 groups according to its proximity to neural niches and to cortex as initially described by Lim DA et al. (14).

Treatment plans were analyzed for each patient and the maximum, minimum and average radiation doses were noted for all of the previously defined volumes. Then a “neural niche” volume was defined as the sum of the SVZ and SGZ volumes, and an average dose was calculated for ipsilateral and contralateral neural niches.

### Clinical and biological variables and follow-up

The following clinical, dosimetric and biological variables were assessed for each patient: age at diagnosis, type of surgery, Mini-Mental State (MMS) and KPS scores, tumor location and PTV. The following molecular variables were also collected: methylation status of the MGMT promoter and Epithelial Growth Factor Receptor (EGFR) amplification (Table 1).

**Table 1:** Patient and tumor characteristics.

	**Number**	**Percentage (%)**
Mean age	56.2 years	
Mean KPS score < 70 >70 unknown	73.27 (50–90) 7 42 1	
**TYPE OF SURGERY**		
Total resection Partial resection Biospy	35 6 9	70 11 19
**MGMT PROMOTER**		
No methylation Methylation Unknown	27 15 8	54 30 16
**EGFR**		
No amplification Amplification Unknown	15 16 19	30 32 38
**LOCATION**		
Frontal Temporal Parietal Occipital	17 18 14 1	34 36 28 2
**MMS**		
Normal Abnormal Unknown	24 24 2	48 48 4
**LIM CLASSIFICATION**		
Zsv–cortex+	9	18
Zsv–cortex−	13	26
Zsv+cortex+	5	10
Zsv+cortex–	17	34
Unknown	6	12
**RECURRENCE**		
No Local Regional Unknown	4 36 6 4	8 72 12 8

Patient follow-up consisted of a clinical examination once a week during radiotherapy then again for each round of chemotherapy, and a MR scan every 2 months. Response and progression were evaluated according to Response Assessment in Neuro-Oncology (RANO) criteria (15).

### Statistical analysis

For each given parameter, two groups were defined according to whether data was below or above the 3rd quartile. All analysis was carried out with a bilateral alpha risk of 5%. Categorical data were expressed as frequency or percentage whereas continuous variables were expressed as median, minimum, and maximum. The *t*-test or Mann-Whitney U test was used to compare quantitative variables. Qualitative variables were analyzed using the Fisher's exact or Chi-square tests. Correlations were studied using Pearson's or Spearman's rank correlation. The length of time until the occurrence of events was estimated using the Kaplan-Meier method and compared with a log-rank test. Overall survival was defined as the length of time between initial surgery and death from any cause, ending on the date of the last given information. As for PFS, it was defined as the length of time between initial surgery and tumor progression or relapse, ending on the date of the last tumor assessment. Univariate and multivariate analyses were used according to Cox's proportional hazards test in order to estimate the Hazard Ratio (HR). We treated dosimetric variables as dichotomous variables using the third quartile as a cut-off. We included in the multivariate analysis all the variables with *p* ≤ 0.1 in the univariate analysis. As a complementary study, RPA was also used to group patients into different categories. The input variables entered in RPA were:

Ipsilateral SVZ mean dose, ipsilateral SGZ mean dose, contralateral SGZ mean dose, contralateral niche mean dose, ipsilateral niche mean dose, total niche mean dose, type of surgery, MGMT status, EGFR status, Lim classification, brain tumor location, GTV, PTV, age, KPS score.

RPA divided patients at each step into two groups based on the covariate that provided maximum separation with respect to prognosis and accounted for interactions between factors. The RPA algorithm is based on the optimized binary partition of these subgroups which enables classification of patients into successively more homogeneous prognostic groups based on multiple input variables. RPART performs a tenfold cross-classification by default to help evaluate the reliability of the tree model. The full sample is randomly divided into 10 sub-samples. Internally, the full RPART tree is carried out with 90% of the full sample, and the remaining 10% of the sample is used as a validation dataset to calculate a cross-classification error rate. This procedure is repeated 10 times, each time with 9 subsets as the modeling dataset and the remaining 1 subset as the validation dataset. Additional technical details can be found in this document: (Therneau TM EJ. An Introduction to Recursive Partitioning Using the RPART Routine. Mayo Clinic, Rochester, MN. 1997) (16).

In this study, we performed RPA using routinely available clinical variables and NSC niche dose-volume parameters to determine risk groups associated with survival. Clinical utility was enhanced by rounding the cut-off points to the nearest significant digit. The OS rates were compared between the RPA risk groups using log-rank tests. The primary endpoint was survival calculated from the date of surgery to the date of either the last follow-up or death using the Kaplan-Meier method. Incomplete or missing data were removed from analysis. Statistical significance was set at *p* < 0.05. Analyses were carried out using the statistics software SPSS version 20®.

## Results

**Figure 2:**
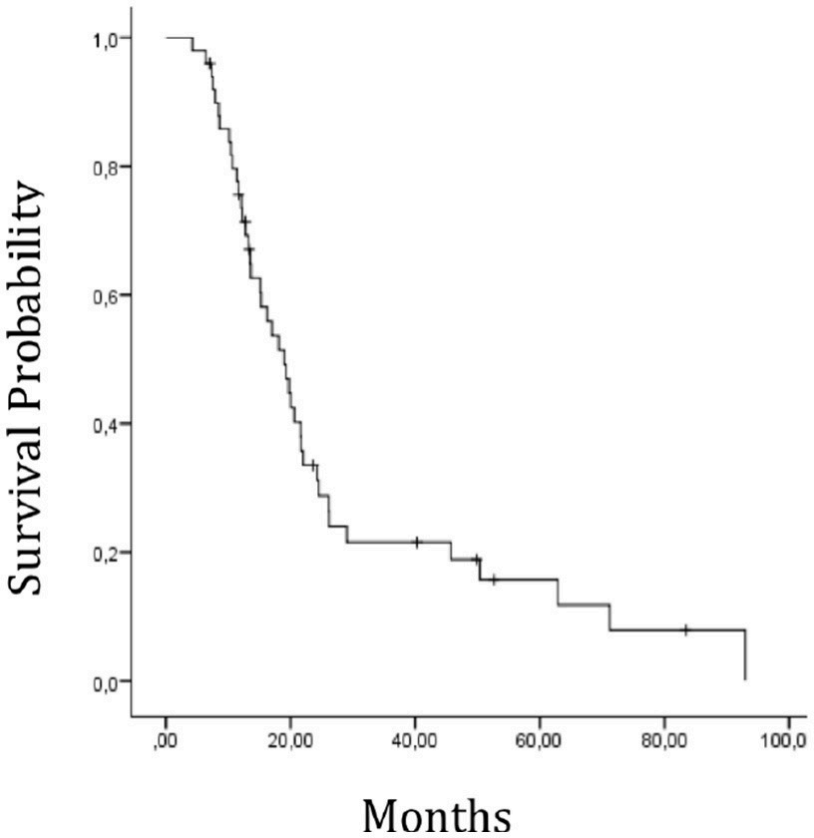
Overall survival for the entire cohorte.

Among the 67 patients for whom a pre-therapy MR scan was available, 3 treatment plans could not be recuperated, 6 patients were missing clinical data, 5 patients had mixed tumors and 3 patients had undergone radiotherapy alone which was hypofractionated. These patients were all excluded. In total 50 patients were retained for the final analysis. Patients and dosimetric characteristics are provided in Tables 1, 2. In the population under study, the mean length of follow-up was 23.8 months (ranges 7.0 to 83.9). Forty-six of the 50 patients relapsed. In 41 cases, this relapse could be characterized as ipsilateral (31 patients) or contralateral (10 patients). Median PFS was estimated at 10 months [IC95 (8.4–11.5)]. Furthermore 41 patients out of 50 had deceased at the time of analysis. The median of OS was estimated at 19.1 months [IC95 (14.6–23.7)] (Figure 2).

**Table 2:** Dosimetric parameters including third quartile (Q3).

	**Median (cGy)**	**(Min–Max) (cGy)**	**Q3 (Gy)**
Ipsilateral SVZ mean dose	5180.95	(2484.60–6067.7)	57.4
Contralateral SVZ mean dose	2662.05	(515.40–5790.60)	40.6
Ipsilateral hippocampus mean dose	5450.05	(400.60–6152.40)	59.04
Contralateral hippocampus mean dose	1755.80	(189.90–5750.00)	33.18
Ipsilateral neural niche mean dose	5314.15	(1721.85–6087.70)	56.97
Contralateral neural niche mean dose	2247.60	(352.65–5591.30)	35.84
Bilateral neural niche mean dose	3711.95	(1072.18–5601.50)	44.06
	Median (cc)		
PTV	306.61		

### Univariate analysis

Among clinical parameters, only the type of surgery (*p* = 0.021) and PTV (*p* = 0.013) were significantly associated with OS. Among dosimetric parameters, the mean dose to the ipsilateral SVZ (*p* = 0.008), to ipsilateral neural niches (SVZ + SGZ) (*p* = 0.004), to the contralateral neural niches (*p* = 0.016), and to bilateral neural niches (*p* = 0.019) were correlated with OS but had no impact on PFS (Table 3).

**Table 3:** Univariate analysis.

	***p***	**HR**	**95% CI**
Age	0.804	0.996	0.963–1.03
KPS score ≤ 70	0.553	0.728	0.255–2.078
Surgery			
Total or partial	**0.015**	2.294	1.174–4.48
MGMT promoter methylation (yes)	**0.048**	0.433	0.189–0.993
EGFR amplification (yes)	0.073	0.484	0.218–1.071
Ipsilateral SVZ mean dose ≥ 57.4 Gy^*^	**0.001**	2.418	1.26–4.63
Contralateral SVZ mean dose ≤ 41.6 Gy^*^	0.289	0.684	0.339–1.38
Ipsilateral neural niche mean dose ≤ 57 Gy^*^	**0.004**	0.350	0.171–0.714
Contralateral neural niche mean dose ≥ 35.8 Gy^*^	**0.075**	0.436	0.222–0.856
Bilateral neural niche mean dose ≥ 44 Gy^*^	**0.019**	0.434	0.216–0.871
PTV	**0.013**		1.001–1.005
Location	ns		
Lim classification	ns		

* *Q3 parameters*.

*SVZ, SubVentricular Zone; PTV, planning target volume*.

### Multivariate analysis

Multivariate analysis revealed only one independent prognostic factor: EGFR amplification (*p* = 0.019) with a trend for type of surgery (*p* = 0.07) and MGMT promoter methylation status (*p* = 0.07) for OS. Again, no factor was associated independently with PFS (Table 4).

**Table 4:** Multivariate analysis.

	***p***	**HR**	**95% CI**	
Total or partial surgery	0.072	2.691	0.915	7.9
MGMT promoter methylation	0.077	0.391	0.138	1.1
EGFR amplification	0.019	0.356	0.150	0.84
Ipsilateral SVZ mean dose (q3)	0.851	1.193	0.189	7.5
Ipsilateral niche mean dose (q3)	0.142	0.377	0.102	1.3
Contralateral niche mean dose (q3)	0.284	2.003	0.563	7.1
PTV	0.317	1.002	0.998	1

*SVZ, SubVentricular Zone; PTV, planning target volume*.

### Recursive partitioning analysis

**Figure 3:**
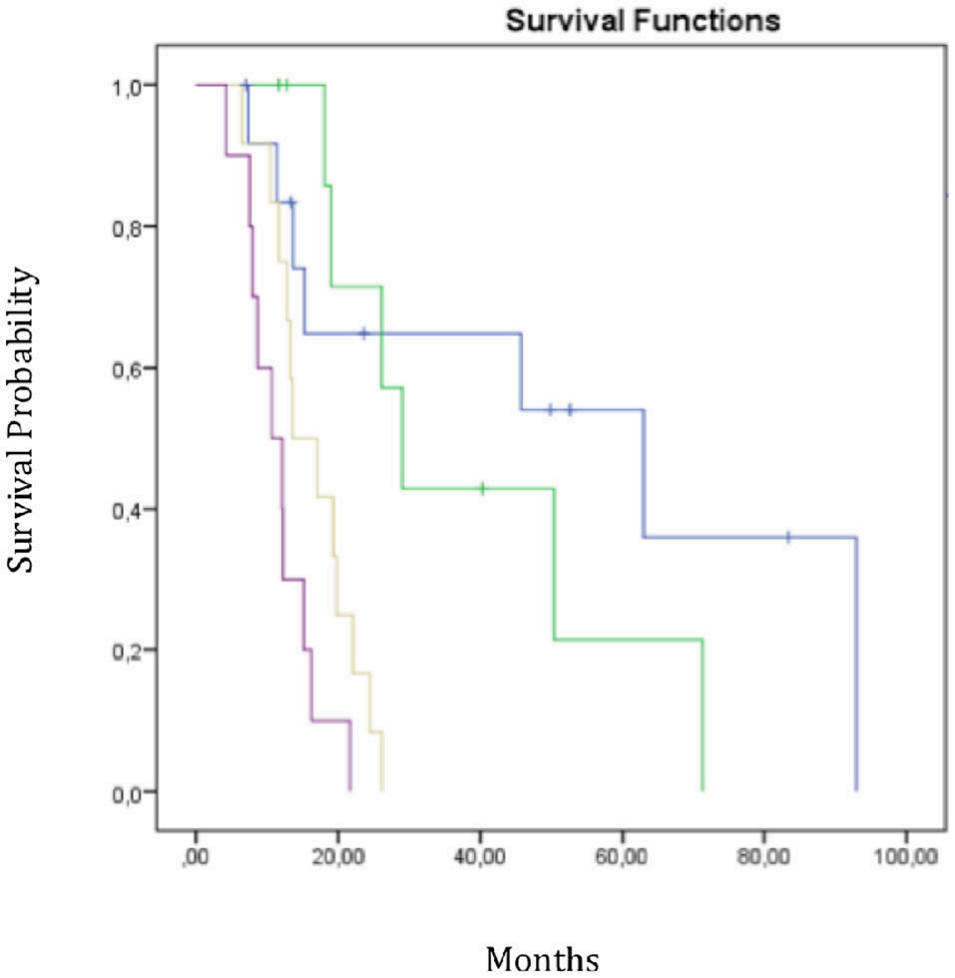
Overall survival curves according RPA subgroups. Group 1 in blue-group 2 in green-group 3 in yellow-group 4 in purple;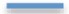 Mean dose homolateral niches < 58 Gy + MGMT methylation;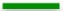 Mean dose homolateral niches < 58 Gy+No methylation + Mean dose contralateral Zsv < 18.6Gy;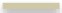 Mean dose homolateral niches < 58 Gy+No methylation + Mean dose contralateral Zsv >18.6Gy;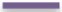 Mean dose homolateral niches >58 Gy.

We performed RPA to establish a new prognostic model for OS to identify predictors of survival and generate a clinical decision tree. As shown in Figure 3, the patients were divided into four risk groups. Patients with a mean ipsilateral neural niche dose below 58.01 Gy and methylated MGMT promoter were stratified into the low-risk group (group 1) (reference). Patients with mean ipsilateral neural niche dose below 58.01 Gy and unmethylated MGMT promoter were subdivided according to mean contralateral SVZ dose into two groups: moderate (group 2) [(mean contralateral SVZ dose < 18.6 Gy); *p* = 0.43] and high risk (group 3) (mean contralateral SVZ dose > 18.6 Gy; *p* = 0.002). Patients with mean ipsilateral neural niche doses above than 58.01 Gy were at very high-risk (group 4) in terms of overall survival (*p* > 0.001) (Table 5).

**Table 5:** Overall survival according to RPA subgroups and log rank-test.

	***P*-value**	**HR (95.0% CI)**
Group	0.000	–
Group 2 vs. 1	0.427	1.591 (0.506– 5.010)
Group 3 vs. 1	0.002	6.340 (1.992–20.178)
Group 4 vs. 1	0.000	13.826 (3.994–47.860)

## Discussion

Despite the multimodality of treatment for glioblastoma, prognosis still remains very poor and this could partly be explained by its biomolecular profile and is probably linked to the involvement of NSC in this disease. Our study reports a significant negative impact of higher doses of radiation received by normal stem cells on OS in a homogeneous subgroup of patients with IDH-wild-type glioblastoma, whilst taking into account MGMT promoter methylation status (3) and EGFR amplification. This is the first study about stem cells dose impact taking into account molecular status in glioblastoma population. This is also the first study to propose a RPA including molecular markers and dosimetric parameters to define risk prognostic groups.

Numerous studies have shown that neural niches can promote tumor development. Following the transformation of NSC into CSC after a punctual mutation, CSC could migrate out of the neural niches to initiate tumor growth (5). Furthermore, the neural niche environment would be ideal for maintaining CSC in a state of hypoxia, therefore encouraging their radioresistance (8), but also ideal for nurturing them via the specific growth factors which can be found around NSC.

Scientific literature reports controversial results. A retrospective study by Evers et al showed the impact of radiation therapy on neural niches for 55 patients treated for diffuse grade III or IV gliomas (10). They reported that a radiation dose > 43 Gy to the bilateral SVZ significantly correlated with better PFS (7.2 vs. 15 months; *p* = 0.03). In their analysis, a dose > 43 Gy to the SVZ appeared to be a positive prognostic factor for PFS (HR = 0.735; *p* = 0.019). This study was the first to show the impact of irradiating neural niches on the survival of patients with glioblastoma. The authors explained that the lack of impact on OS was due to the multiple salvage therapies used at relapse and suggested, in order to defend their results, a sensitivity of stem cells to low fractionated doses (17).

In 2013 the same team studied a multicentre cohort of 173 patients and reported that radiation doses > 59.4 Gy to the SVZ correlated with better PFS (12.6 vs. 9.9 months; *p* = 0.042) confirmed using multivariate analysis (HR = 0.45; *p* = 0.009) (11). However, the lack of molecular variables and the comparison of two unequal populations (21 patients receiving the high ipsilateral SVZ dose vs. 152 patients receiving the lower ipsilateral SVZ dose) again make these results difficult to interpret. Furthermore, Gupta et al. showed that, for 40 patients studied, a high radiation dose to the ipsilateral SVZ seemed to be a positive prognostic factor for OS [HR = 0.87; IC95 (0.77; 0.98)] but without impact on PFS in multivariate analysis (12). Similar correlations were reported in glioblastoma populations regarding radiation doses higher than 40 or 50 Gy to ipsilateral neural niches respectively by Chen et al. and Kusumawidjaja et al.

Nevertheless, the fact that all these cohorts were heterogeneous with grade III and IV diffuse gliomas and/or that molecular profiles such as MGMT promoter methylation or IDH1/2 mutations were not taken into consideration, all represent limits that make the results difficult to interpret and compare (18, 19).

In our study that focussed on IDH-wild-type glioblastoma, we found that radiation doses below average to ipsilateral SVZ (< 57.4 Gy), to ipsilateral neural niches (< 57 Gy) and to contralateral neural niches (< 35 Gy) had a significant positive impact on OS with medians (*p* = 0.001), (*p* = 0.015), and (*p* = 0.029) respectively, but no impact however on PFS. The coherence of our results is backed by published fundamental studies.

Neural niches form an ideal environment, perfectly adapted to CSC survival. Some *in vitro* and *in vivo* studies have already shown that CSC were particularly radioresistant (6, 7, 6, 20), especially when they were in a hypoxic condition such as that found in neural niches (9). On the contrary, NSC seem to have very different characteristics and could even be specifically sensitive to ionizing radiation (21). Even though these studies are limited to being done *in vitro* or *in vivo*, they highlight a difference in sensitivity which could help understand the different results. Thus, when high doses are delivered to neural niches, perhaps the NSC are eliminated without however destroying the CSC. Thus, the equilibrium between NSC-CSC would be disrupted and the neural niche could be recolonized exclusively by CSC, as has been shown by some mathematical studies (6), which could explain the decrease observed in OS. Finally, it seems more than probable that certain subgroups whose tumorogenesis originates from stem cells would suffer from irradiation of neural niches.

Several authors have reported also similar clinical results. Achari et al. reported in a series of 61 glioblastoma patients that a median dose higher than 55.2 Gy to ipsilateral neural niches was associated with significant lower PFS (0.013) and OS (*p* = 0.028). In multivariate analysis, temozolomide compliance, MGMT promoter methylation status, ipsilateral neural niche median dose and ipsilateral SVZ median dose were independent prognostic factors (*p* < 0.001, 0.011, 0.017, 0.017, respectively) (22). Elicin et al. reported that a higher dose than 62.25 Gy to ipisilateral neural niches was associated with a poor OS in the same cohort of patients (23).

In this current study, we used RPA to integrate more significantly prognostic variables such as molecular and dosimetric parameters and to avoid subjective selection of variables. Such an approach has been developed in 2 studies to stratify glioblastoma patients, one from TCGA and second from RTOG 0525 datasets with the incorporation of molecular prognostic markers. In these 2 analyses, MGMT promoter methylation status and MGMT protein expression participated in RPA model prediction (24, 25). We identified three significant prognostic factors: first split with average ipsilateral neural niche doses below 58 Gy, then second split with methylated MGMT promoter and last split with average ipsilateral SVZ doses < 18.6 Gy.

If some other authors have already reported a negative survival impact of high doses to stem cells, to our knowledge, this is the first time that dosimetric parameters related to neural niches were identified as significant prognostic factors by RPA in well-defined population of *de novo* glioblastoma.

The limits of our study are the fact that it is a retrospective study with unknown bias and that our sample number remains small. On the other hand, our population is homogeneous in terms of IDH status and treatment modalities and our study takes into account molecular data associated with survival such as MGMT promoter methylation status and EGFR amplification.

## Conclusion

In this study, high radiation doses to neural niches are a pejorative prognostic factor for patients treated for IDH-wild-type glioblastoma. Using RPA we identified 4 prognostic groups of patients according to MGMT promoter methylation status, maximum doses delivered to the ipsilateral NSC and contralateral SVZ. This result contributes to the foundation of knowledge on stem cells radiation.

These results must be confirmed in a larger series of patients and then they could be added to dosimetric decision-making in glioblastoma patients.

## Ethics statement

This study was carried out in accordance with the recommendations of EANO guidelines. The protocol was approved by the local ethic committee. All subjects gave written informed consent in accordance with the Declaration of Helsinki.

## Author contributions

XM: Idea conception, data collection, dosimetry making, paper writing; WE and ET: Data collection, paper writing; AB: MGMT status reviewing for each tumor; GP and OC: Paper writing; TH: Data collection; LS: Dosimetry making and reviewing for data collection; NM and DF-B: Molecular status reviewing for each tumor; LP: Idea conception, data collection, dosimetry making, paper writing. MB: Performed all the statistical analysis.

### Conflict of interest statement

The authors declare that the research was conducted in the absence of any commercial or financial relationships that could be construed as a potential conflict of interest.

## Abbreviations

NSC: neural stem cell
SVZ: SubVentricular Zone
SGZ: Sub Granular Zone
INMD: ipsilateral NSC mean dose.
